# Auditory evoked BOLD responses in awake compared to lightly anaesthetized zebra finches

**DOI:** 10.1038/s41598-017-13014-x

**Published:** 2017-10-19

**Authors:** L. Van Ruijssevelt, J. Hamaide, M. T. Van Gurp, M. Verhoye, A. Van der Linden

**Affiliations:** Bio-Imaging lab, Department of Biomedical Sciences, University of Antwerp, 2610 Wilrijk, Belgium

## Abstract

Functional magnetic resonance imaging (fMRI) is increasingly used in cognitive neuroscience and has become a valuable tool in the study of auditory processing in zebra finches, a well-established model of learned vocal communication. Due to its sensitivity to head motion, most fMRI studies in animals are performed in anaesthetized conditions, which might significantly impact neural activity evoked by stimuli and cognitive tasks. In this study, we (1) demonstrate the feasibility of fMRI in awake zebra finches and (2) explore how light anaesthesia regimes affect auditory-evoked BOLD responses to biologically relevant songs. After an acclimation procedure, we show that fMRI can be successfully performed during wakefulness, enabling the detection of reproducible BOLD responses to sound. Additionally, two light anaesthesia protocols were tested (isoflurane and a combination of medetomidine and isoflurane), of which isoflurane alone appeared to be the most promising given the high success rate, non-invasive induction, and quick recovery. By comparing auditory evoked BOLD responses in awake versus lightly anaesthetized conditions, we observed overall effects of anaesthetics on cerebrovascular reactivity as reflected in the extent of positive and negative BOLD responses. Further, our results indicate that light anaesthesia has limited effects on selective BOLD responses to natural versus synthetic sounds.

## Introduction

Functional magnetic resonance imaging (fMRI) is currently one of the most important neuroimaging methods in cognitive neuroscience^1^. FMRI infers brain activity and function based on changes in local haemodynamics in humans and in a wide variety of animal models. The main advantages of this technique are its non-invasive nature and its whole brain approach. Conversely, one of its greatest limitations is that subjects must remain immobilized throughout the scanning procedure for fMRI to be successful. For animals, this usually requires the use of anaesthesia. However, anaesthetic agents can interfere with normal brain function and often limit the type of experiments that can be performed. Therefore, an increasing number of studies have explored possible ways of performing fMRI studies in awake animals. Different protocols have been proposed ranging from paralyzing the animals with curare-like drugs^2^ to the use of special restraining devices combined with an acclimation procedure to accustom subjects to the imaging protocol (e.g.^3–5^). For ethical reasons, the latter method is usually preferred.

fMRI has also been implemented in the study of zebra finches, a songbird species recognized as the most suitable animal model currently available for studying vocal learning and auditory processing of learned complex vocalizations^6,7^. To date, fMRI studies in this model are limited to the study of auditory processing in anaesthetized animals. However, the feasibility of fMRI in awake zebra finches has not yet been explored. The possibility of performing fMRI during wakefulness would open a wide variety of new avenues to study brain function in this animal model. Performing fMRI under awake conditions would allow more advanced sensory stimulation and the possibility to image cognitive processes like learning and association processes. Also, the study of the influence of pharmacological agents on normal brain function would become possible without the potential for confounding effects caused by the interaction between the agent and the anaesthetic. In addition, the results obtained from these experiments would be more comparable to findings in human fMRI studies, which are usually performed under awake conditions. Thus, the primary goal of this study was to explore the feasibility of performing fMRI in awake zebra finches using restraint combined with an acclimation procedure to reduce stress caused by the restraining and scanner noise.

Even with the availability of procedures for fMRI in awake subjects, in some cases anaesthesia may be preferable. For example, in studies of very young animals, longitudinal studies, or studies in large groups of subjects, acclimation can be too time consuming or impractical. In such cases, it is important to have reliable and robust anaesthetic protocols available and to know the effects of these anaesthetics on the BOLD response. Consequently, the second goal of this study was to test two light anaesthesia protocols for zebra finch fMRI experiments and compare BOLD responses to auditory stimulation obtained in anaesthetized birds to those in awake birds. As such, we aimed to verify the effects of light anaesthesia on the BOLD response, specifically for auditory processing in the zebra finch auditory forebrain. Two different anaesthetics were included: (i) isoflurane and (ii) medetomidine. These agents have been previously used in auditory fMRI experiments in zebra finches^8–12^ and are widely used in fMRI studies in other animal models^13,14^. In contrast to earlier studies, the tested doses were considerably lower, especially for medetomidine. At the low doses used, animals were lightly anaesthetized evident by the presence of reflexes including the pedal reflex. During the induction phase of both protocols the anaesthesia level was higher to allow proper positioning of the animals in the scanner bed. Based on reports by Grandjean and colleagues, medetomidine was combined with a very low concentration of isoflurane (vasodilator) to counterbalance its vasoconstrictive effect^15^. We further refer to this anaesthesia protocol using the ‘Med/Iso’ acronym.

## Results

### Feasibility of fMRI in awake zebra finches

#### Success of the acclimation procedure

**Figure 1:**
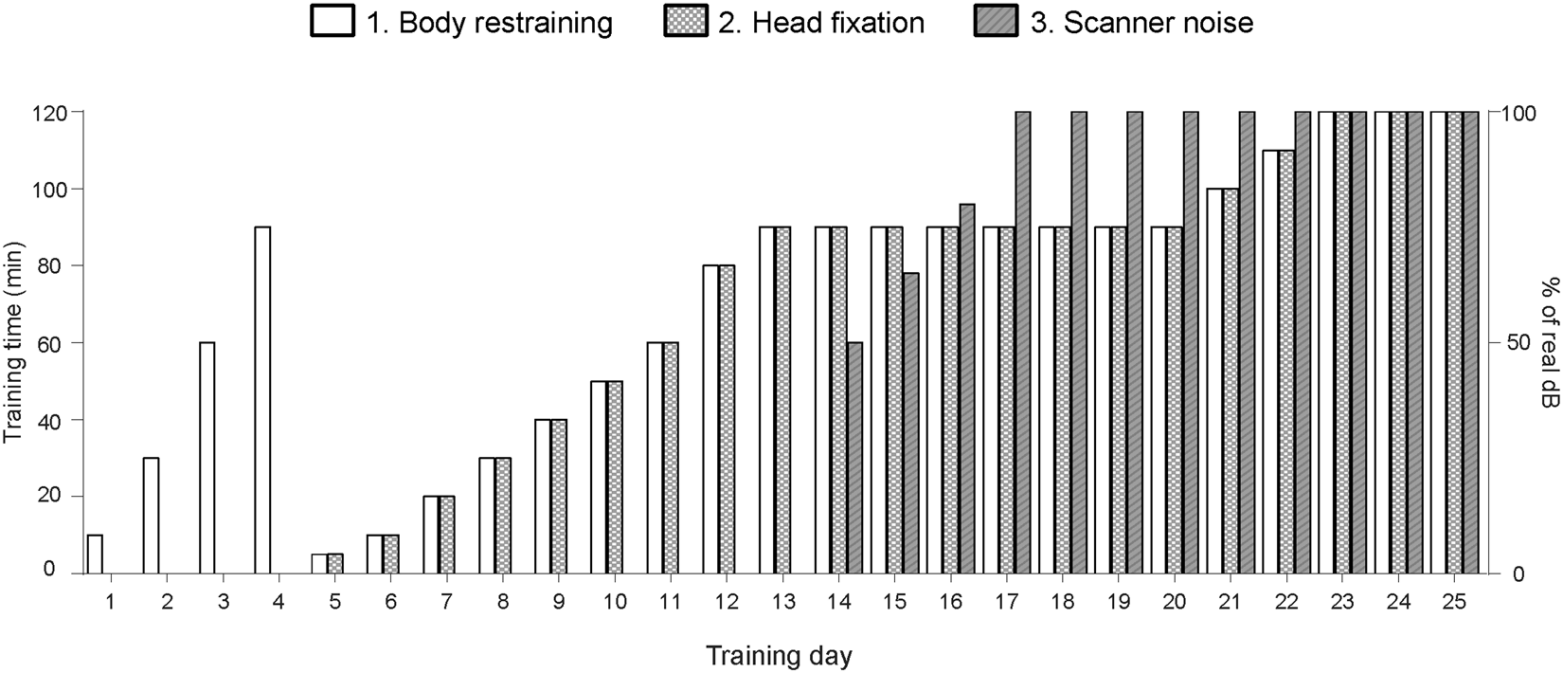
Schematic representation of the acclimation procedure.

The main challenge of performing fMRI in awake animals is to minimize head motion during scanning. In this study, performed in 12 adult male zebra finches, we restrained the birds to overcome this issue and subjected them to an acclimation procedure to habituate them to restraint and scanner noise. The acclimation procedure was based on a protocol which was previously applied to train pigeons to be scanned in awake conditions^16^. The procedure consisted of 3 consecutive phases: (1) Acclimation to body restraining in a dark scanner-like environment; (2) Acclimation to total restraint including head fixation; (3) Acclimation to total restraint and scanner noise (Fig. 1). In a small minority of the animals (n = 2) the acclimation was not successful. In these animals, we observed excessive stress evident by a persistent struggle to escape and a very high breathing rate ( > 150 breaths/minute). Consequently, these birds were not subjected to the entire imaging protocol and thus excluded from the study. In the other animals (n = 10), no obvious visual signs of distress were present during scanning, indicating that the acclimation procedure was successful. Maximal head motion throughout the time of fMRI scanning in these animals was small (maximal translation: 0.106 ± 0.024 mm; maximal rotation: 0.0125 ± 0.0019) resulting in high quality fMRI images.

#### Head motion

**Figure 2:**
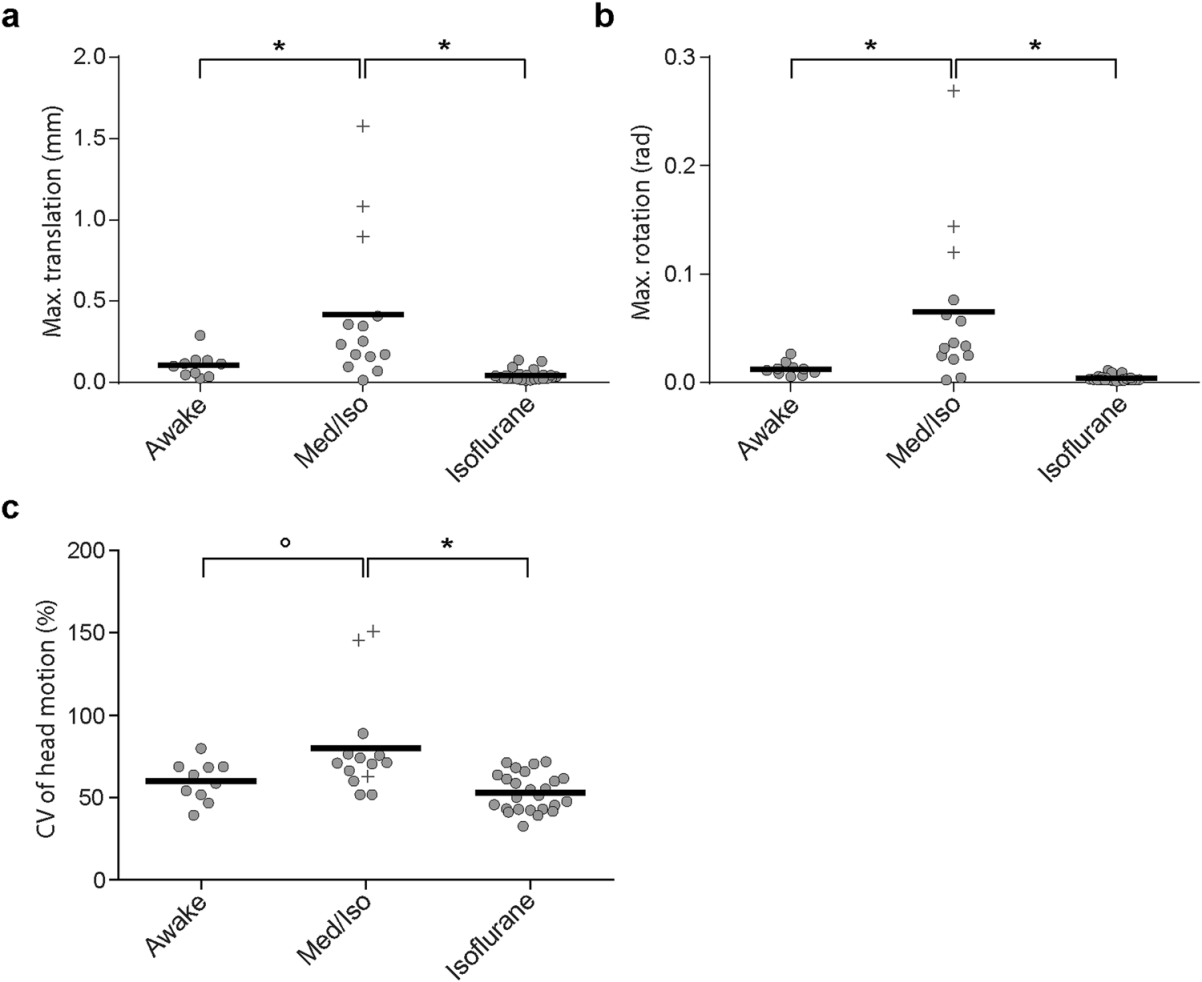
Head motion during fMRI scanning in awake and anaesthetized conditions. The graphs report the maximal head translation (**a**) and rotation (**b**), and the coefficient of variation (CV) of the total head motion over time (**c**) for the fMRI scans of awake birds and birds anaesthetized with a low dose of Med/Iso or isoflurane. Individual measurements are depicted by grey circles and the group mean by the black line. Crosses point to three animals of which the maximal translation was beyond 2 voxel sizes and which were therefore excluded from the subsequent analyses (*p < 0.05).

Limited head motion during scanning reflected the success of the acclimation procedure for awake fMRI. Comparison of the maximal head translation and rotation, as well as of the coefficient of variation (CV) of the total head motion (standard deviation normalized to the mean head motion over time) for the fMRI time series acquired during wakefulness and under light anaesthesia revealed a significant main effect of condition for maximal translation and rotation (*F*_*2*,*35*.*9*_ = 10.4850; *p* = 0.0003 and *F*_2,35.7_ = 11.3693; *p* = 0.0002 respectively) as well as for the CV of the total head motion (*F*_*2*,*36*.*3*_ = 7.8499; *p* = 0.0015). Maximal head translation and rotation were not different during wakefulness vs isoflurane anaesthesia (*p* = 0.7824 and *p* = 0.8291 respectively), but were significantly higher under Med/Iso anaesthesia (Max. translation: awake vs Med/Iso *p* = 0.0142; isoflurane vs Med/Iso *p* = 0.0002; Rotation: awake vs Med/Iso *p* = 0.0001; isoflurane vs Med/Iso *p* = 0.0080; Fig. 2a,b). The analysis of the CV of the total head motion over time revealed similar trends, namely that the head motion did not differ for scans acquired during wakefulness versus anaesthesia (*p* = 0.6433) and that head motion was higher for scans acquired under Med/Iso anaesthesia (awake vs Med/Iso *p* = 0.0558; isoflurane vs Med/iso *p* = 0.0010) (Fig. 2c). Three animals measured under Med/Iso anaesthesia showed translation beyond 2 voxels (> 0.5 mm) and were therefore excluded from the subsequent analyses. One additional animal measured under Med/Iso anaesthesia was excluded from this group because it repeatedly woke up within minutes after induction of the anaesthesia.

#### Physiological parameters

Besides limited head motion, stability in physiological parameters such as breathing rate and body temperature is important for successful fMRI measurements. Breathing rate was measured using a pressure pillow placed under the bird’s chest. In general, we observed stable levels for the breathing rate in all conditions. To quantify respiratory stability during fMRI, we calculated the CV of the subject’s breathing rate by normalizing the standard deviation to the mean breathing rate measured during each fMRI session. The intra-session CV for fMRI acquired under anaesthesia was low (average CV: isoflurane 4.9 ± 1.7%; Med/Iso 6.7 ± 5%) and comparable for Med/Iso and isoflurane (*F*_1,12.8_ = 1.3887; *p* = 0.2601) (Supplementary Fig. S1). Additionally, we observed that the average breathing rate was significantly lower under isoflurane anaesthesia (69.588 ± 27.296 breaths/min) relative to Med/Iso anaesthesia (97.985 ± 37.477 breaths/min) and awake conditions (114.05 ± 34.10 breaths/min) (Supplementary Fig. S1). During scanning under anaesthesia, body temperature was controlled by a feedback controlled air system which kept the temperature within a narrow range of 40.0° ± 0.1 at all times.

### Success rate of fMRI in awake and anaesthetized conditions

During fMRI scanning, the animals were exposed to three different types of auditory stimuli: synthetic pure tones (PT), unfamiliar conspecific song (CON), and heterospecific song (HET). These different types of stimuli were presented in random order following an ON-OFF block paradigm. Datasets which showed limited head motion and a clear increased blood-oxygen-level dependent (BOLD) signal in the primary auditory region Field L in response to sound (independent of the type of stimulus) were included in the subsequent group analysis. In the awake condition, all 10 animals showed field L activation in response to sound. On the other hand, two animals measured under Med/Iso anaesthesia and three animals measured under isoflurane anaesthesia showed no clear BOLD response within the auditory forebrain to any of the stimuli. These animals were, in addition to the animals with excessive head motion, excluded from further analysis. Accordingly, the overall success rate of fMRI was 60% (9/15 animals) under Med/Iso anaesthesia, 80% (10/12 animals) during wakefulness after acclimation, and 88% (22/25 animals) under isoflurane anaesthesia (see Supplementary Table S2 for an overview).

### Differences in BOLD responses to auditory stimulation during wakefulness and under light anaesthesia

**Figure 3:**
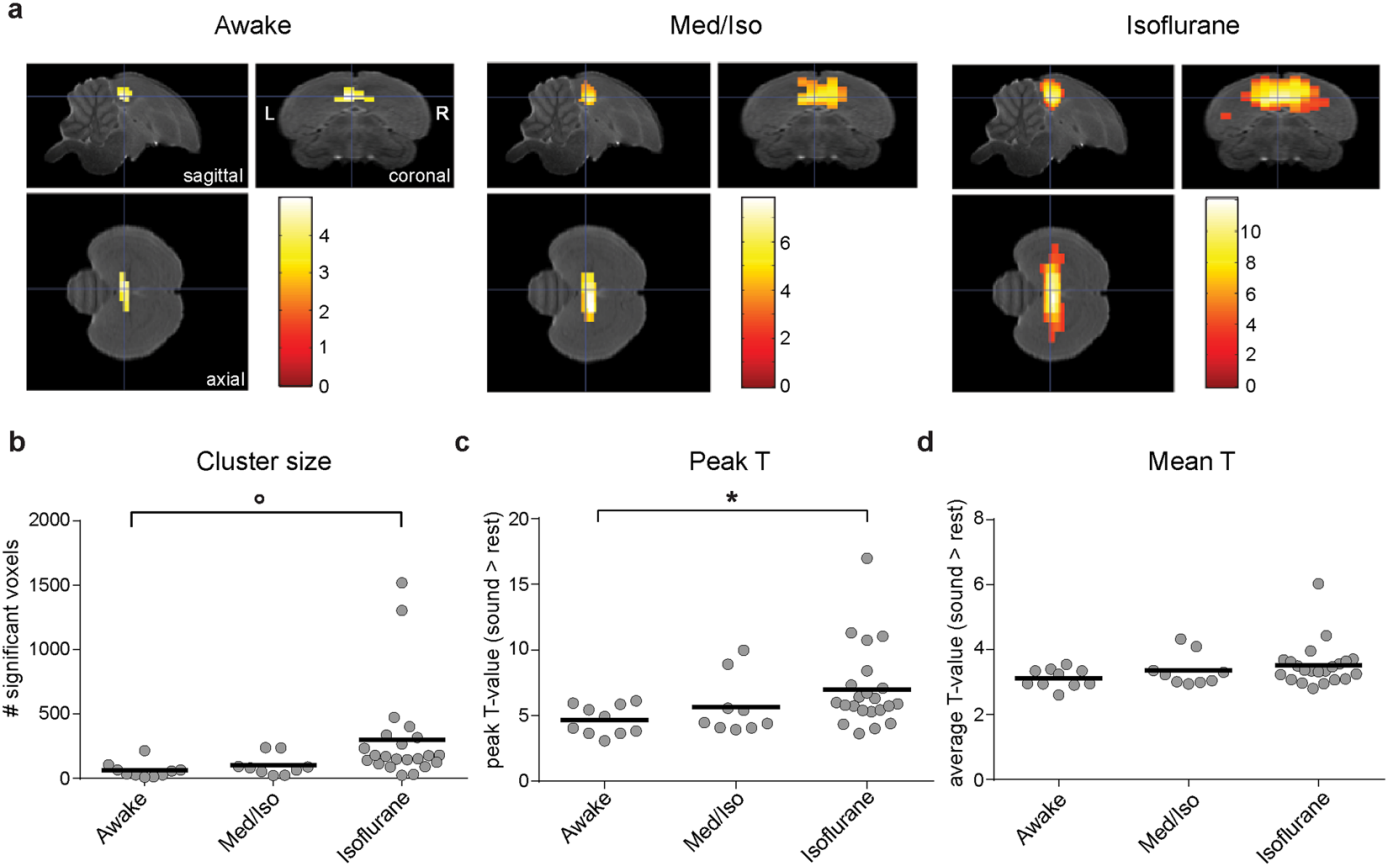
BOLD responses in the zebra finch auditory forebrain in response to sound (vs rest) for the different conditions. (**a**) The images represent results of the voxel based group analysis for all sounds > rest (one-tailed t-tests) in awake, Med/Iso anaesthetized or isoflurane anaesthetized conditions. *T*-values are colour coded according to the scale next to the figures and only voxels with *T*-values > 3.32 (*p*_uncorrected_ < 0.001) are displayed. The statistical maps are superimposed on images from the population based template. (**b**–**d**) The graphs illustrate the cluster size (**b**), peak *T* (**c**) and mean *T* (**d**) of the activated cluster within the auditory forebrain in response to sound in awake, Med/Iso anaesthetized and isoflurane anaesthetized conditions. Individual measurements are depicted by grey circles and the group mean by the black line (*p < 0.05; °*p* < 0.10).

As illustrated in Fig. 3, the voxel-based group analysis revealed a bilateral cluster of activated voxels in response to all stimuli encompassing the primary auditory region Field L and parts of the adjacent secondary regions caudomedial nidopallium (NCM) and caudomedial mesopallium for all conditions. The extent of the activated cluster and the amplitude of the BOLD signal, however, differed depending on the condition. We quantified this difference by extracting the number of voxels within the activated cluster (one-tailed one sample *T*-test: all sounds > rest) and its corresponding peak and mean *T* values for each individual animal. The results of the mixed effect analysis confirmed the different responses to sound for the conditions as presented in the statistical maps in Fig. 3a. A main effect of condition was found for peak *T*-value (*F*_2,16.7_ = 5.7429; *p* = 0.0126) and pointed towards a trend for cluster extent (*F*_2, 19.5_ = 3.1387; *p* = 0.0658) (Fig. 3b,c). The trend for the cluster extent suggested that cluster size was larger under isoflurane anaesthesia compared to awake conditions (isoflurane vs awake *p* = 0.0935). Two animals within the isoflurane anaesthetized group had a cluster extent that was almost 8 times larger than the average cluster extent for the rest of the animals measured under isoflurane anaesthesia (Fig. 3b). These cluster extent data could be considered outliers and thus excluded from the analysis. Doing so reveals a significant difference in cluster size between the conditions (*F*_2, 26.5_ = 8.6023; *p* = 0.0013) with cluster extents which are largest for scans acquired under isoflurane anaesthesia, intermediate for scans acquired under Med/Iso anaesthesia and smallest for scans acquired in awake conditions (isoflurane vs. awake *p* = 0.0015; isoflurane vs. Med/Iso *p* = 0.0942; Med/Iso vs. awake *p* = 0.4846). The peak *T*-value appeared higher for all sounds vs rest under isoflurane anaesthesia as compared to awake conditions (*p* = 0.0129) (Fig. 3c).The mean *T* over all voxels within the activated cluster was, however, not different between the conditions (*p* > 0.10) (Fig. 3d). For the latter analysis of peak *T* and mean *T* as well as for all further reported analyses, the two animals with very large cluster sizes were not treated as outliers and thus included in the analyses. Yet, we verified the effect of excluding them as outliers and found that results were comparable to those reported here.

**Figure 4:**
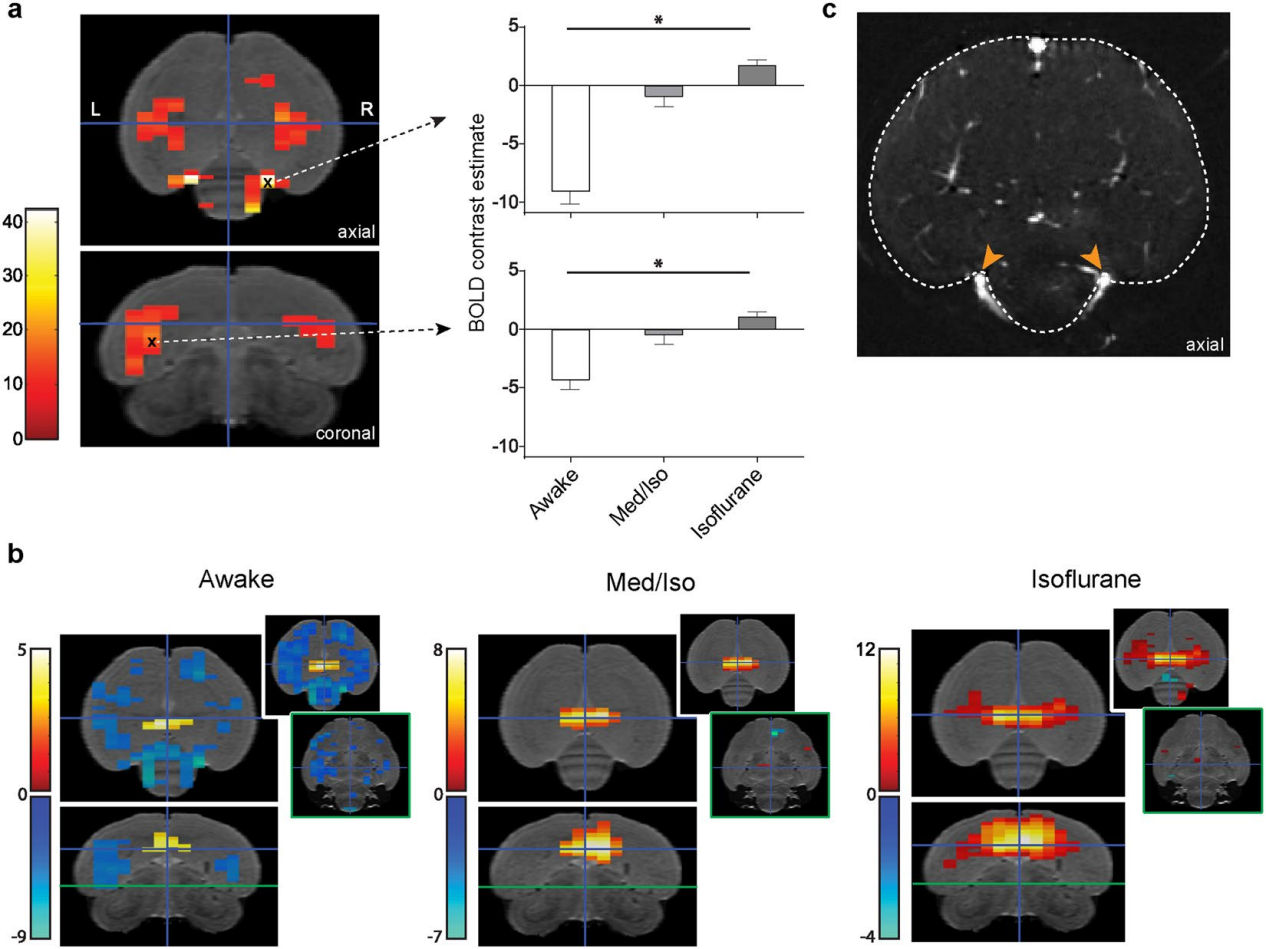
Comparison of the BOLD response to auditory stimulation in awake vs anaesthetized conditions. (**a**) Main effect of condition. (**b**) Detailed graphs for 2 individual voxels within the main effect (voxels marked in (**a**) with ‘x’). The bars represent the mean BOLD response amplitude ( ± SEM) for all stimuli vs rest in the different conditions. (**b**) Positive (red/yellow) and negative (blue/green) BOLD responses to all stimuli in the different conditions. The statistical maps in (**a**) and (**b**) are superimposed on coronal and axial slices of the population based template. Only significant voxels (*p*_uncorrected_ < 0.001; cluster extent >12 voxels) are displayed in the main figures. The insets in (**b**) represent statistical maps at a lower threshold (*p*_uncorrected_ < 0.01; no cluster extent threshold) at two different levels along the dorso-ventral axis as indicated in the coronal figure by the blue and green horizontal lines. (**c**) Representative angiogram of the zebra finch brain. The arrowheads point to large blood vessels near the cerebellum at the location where the peak of the main effect of condition as represented in (**a**) was found.

To further explore the main effect of condition in the voxel based group analysis, we used a whole-brain one-way ANOVA comparing the BOLD response to sound (all stimuli vs rest) over the different conditions. This analysis revealed distinct regions located outside of the previously shown activated area with significant differences in BOLD response to the different conditions (*p*_uncorrected_ < 0.001; clusters >12 voxels following Monte Carlo simulation α < 0.05^17^). No obvious differences in BOLD responses were thus found within the auditory forebrain itself (Fig. 4a). Further testing revealed that in the regions demonstrating a significant condition effect, the BOLD response to sound in the awake condition was negative (Fig. 4a,b). Such negative BOLD responses were not found in the fMRI data acquired under isoflurane anaesthesia and only to a very small extent in the data acquired under Med/Iso anaesthesia (Fig. 4b). Interestingly, by comparing the resulting statistical map of the effect of condition with an angiogram (Fig. 4c), we observed that the voxels with the highest *F* values for the main effect (*F* = 41.9527, *p* < 0.001) matched nearly perfectly with the location of large blood vessels surrounding the cerebellum. This result indicates that a vascular effect might lie at the basis of the observed negative BOLD responses in awake subjects and of the difference in BOLD response between the different conditions.

### Influence of anaesthesia regime on neural selectivity for natural song in the auditory forebrain

**Figure 5:**
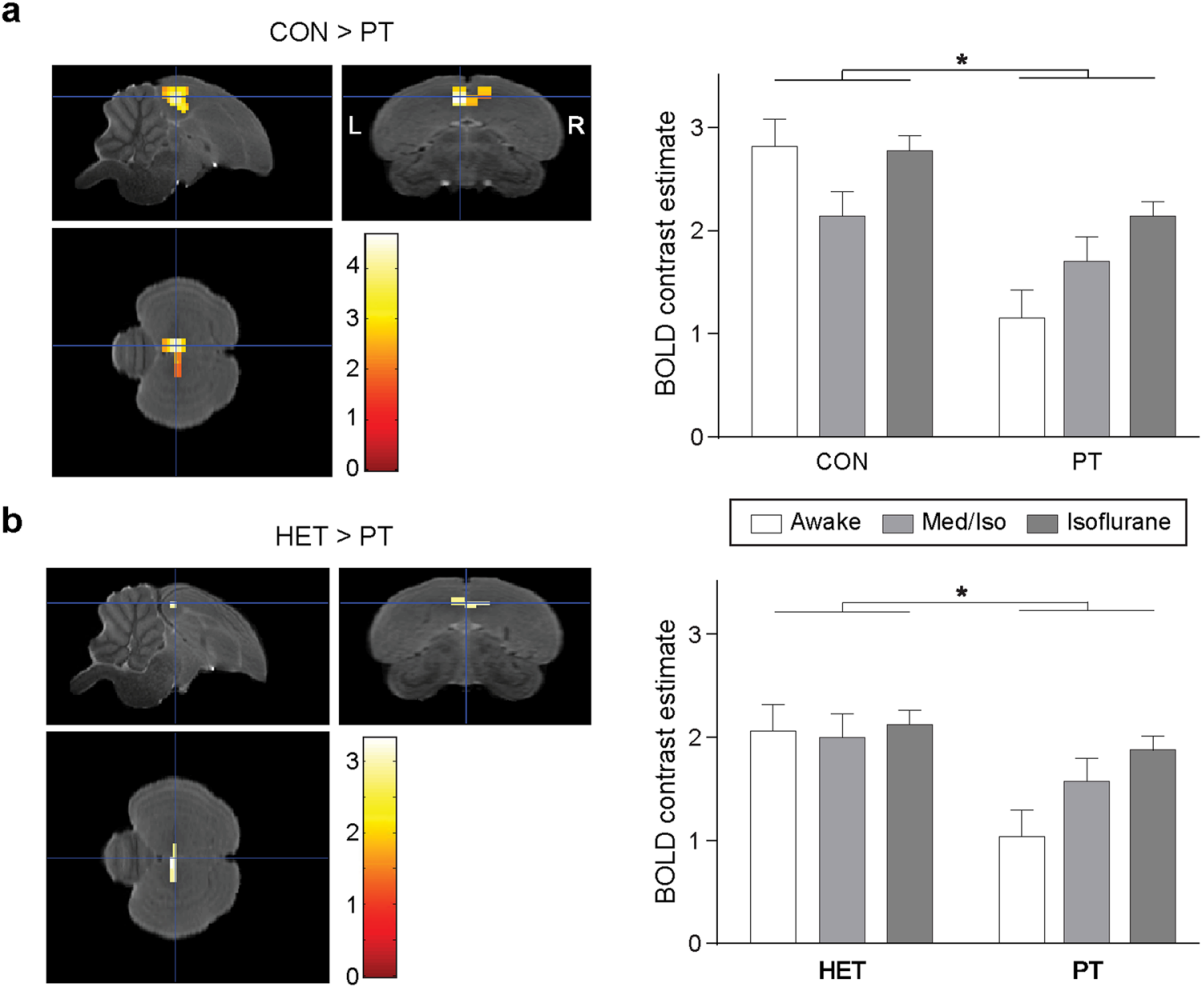
Conspecific and heterospecific song selectivity in the auditory forebrain. The images on the left represent statistical maps for the main CON > PT (**a**) and HET > PT (**b**) selectivity over all conditions superimposed on images of the population based template. Only voxels with a *T* value > 1.66 (*p*_uncorrected_ < 0.05) are displayed. Graphs on the right show the relative response amplitude of responses for the local peak voxel. The zero level corresponds to the estimated mean during rest periods and the error bars to standard errors across subjects (*p_FWE_ < 0.05).

Next, we verified whether selective responses to song within the auditory forebrain were influenced by the condition of the animal. The two-way mixed effect Anova with condition (awake, isoflurane, Med/Iso) as between-subject factor and stimulus (CON, PT, HET) as within subject factor revealed no interaction between stimulus and condition suggesting that the type of anaesthesia or the level of wakefulness of the animal had no effect on song selectivity in the auditory forebrain in this study. Further, for the main effect of stimulus, we found a significant cluster encompassing parts of Field L, CMM and NCM in both hemispheres (*p*_uncorrected_ < 0.05; cluster >53 voxels following Monte Carlo simulation α < 0.05). Post hoc analysis was subsequently performed within this cluster. Family wise error (FWE) correction was applied at the peak voxel level to correct for multiple comparison. This analysis revealed significant selectivity for CON > PT (*T*_max_ = 4.01 i.e. voxel representing the maximal *T*-value among all significant voxels of the cluster; *p*_FWE_ < 0.001) (Fig. 5a) and HET > PT (*T*_max_ = 3.25; *p*_*FWE*_ = 0.040) (Fig. 5b). We also found a trend for CON > HET selectivity in sub-regions of the left auditory forebrain (*p*_uncorrected_ = 0.005) but this selectivity appeared not significant after FWE correction.

## Discussion

The first goal of this study was to examine the feasibility of performing fMRI in awake zebra finches. We tested an acclimation procedure to habituate the animals to total restraint in a noisy scanner environment and showed that by the end of the acclimation period, the subjects could be successfully scanned during a prolonged scanning session with minimal movement and without any visual signs of excessive stress. The success rate of the acclimation procedure was 80%. Successful scanning sessions in acclimated subjects resulted in high quality (f)MRI scans and detection of reproducible BOLD responses to acoustic stimulation in the auditory forebrain. We trained animals over a period of 25 days for restraint during a scanning session of up to two hours, but we are convinced that the length of the acclimation procedure can be reduced for shorter scanning sessions. For a restraining period of up to 90 minutes, for example, the duration can be easily shortened to 20 days. In an earlier study in pigeons, animals were habituated in approximately 17 days to a one hour scanning session using a similar acclimation procedure^16^. We should note that the latter study used a surgically implanted head post to minimize head motion whereas we attained similar results with a non-invasive restraining procedure. Additionally, in contrast to acclimation procedures often applied to rodents (e.g.^4,5,18,19^), no anaesthetic or sedative agents were necessary to place the animal in the restrainer. As such, possible confounding effects caused by the use of anaesthesia, or by excessive stress or pain due to invasive procedures, are completely eliminated with the proposed protocol.

Two light anaesthesia protocols were tested. First, we included the inhalant anaesthetic isoflurane, which is currently the anaesthesia of choice for fMRI in zebra finches^11,12,20–22^. We used a concentration of 1.2% (relative to the carrier gas composed of a mixture of oxygen and nitrogen), which is, according to our experience, the lowest concentration at which adult zebra finches remain still in the scanner and reproducible functional scans can be acquired. Second, the injectable anaesthetic agent medetomidine was included. This anaesthetic has proven valuable in earlier fMRI studies in songbirds^10,23^. Again, we administered the minimal dose – up to 10-fold lower than the dose used in earlier studies – necessary to ensure light anaesthesia and minimize effects on the BOLD response. Based on a study by Grandjean and colleagues in mice, medetomidine was combined with a very low dose of isoflurane to counterbalance its effects on the vascular system; it has been shown that medetomidine induces vasoconstriction, whereas isoflurane causes vasodilatation^15^. To our knowledge, this is the first report on the application of the Med/Iso combination in avian species. Maintaining stable physiological parameters is of utmost importance for generating reproducible fMRI results^24^. A feedback-controlled heating system ensured stable body temperature throughout scanning. Breathing rate was also monitored and appeared, as indicated by the low intra-session CV values, stable throughout scanning under both isoflurane and Med/Iso anaesthetized conditions. When comparing fMRI sessions acquired in awake, Med/Iso- and isoflurane-anaesthetized animals, we observed that the success rate with Med/Iso anaesthesia was remarkably lower relative to the other conditions. The main reason for this low success rate was excessive movement during or prior to scanning, indicating an inadequate level of anaesthesia. Additionally, head motion in the Med/Iso group was overall higher than head motion measured under isoflurane anaesthesia or in awake acclimated conditions. This supports our conclusion that the anaesthesia level obtained with the Med/Iso combination was not sufficient to guarantee a stable level of anaesthesia and to limit head motion without the need for acclimating the animals to the imaging procedure or for tight restraint as was done for the awake animals. Unstable and irreproducible levels of anaesthesia can be the source of great intra-animal variation in functional studies and is thus not recommended for fMRI experiments. Injectable anaesthetics are known to be more prone to inducing irregular levels of consciousness, especially at low doses^25^. With inhalant anaesthesia like isoflurane, the stability of anaesthetic depth can be controlled more easily. Besides stable animal conditions, isoflurane offers two additional major advantages over medetomidine: (i) non-invasive induction, and (ii) fast recovery. Indeed, animals anaesthetized with isoflurane recover quickly within minutes after the experiment without the need of an antidote. The stability of anaesthetic depth coupled with non-invasive induction and quick recovery make isoflurane the best suited anaesthesia for (longitudinal) fMRI studies in zebra finches.

The establishment of a protocol to perform fMRI in awake zebra finches enabled us to compare fMRI scans acquired during wakefulness with scans acquired under light anaesthesia. In doing so, we could verify the effect of low doses of isoflurane and Med/Iso on the BOLD signal. The most striking difference between BOLD responses measured in anaesthetized vs awake birds in this study were the differences in the extent for both the positive and negative BOLD signals. In awake animals, the cluster extent for the positive BOLD response in the auditory forebrain was smallest and a widespread negative BOLD signal was found in the regions surrounding this activated area. This negative BOLD signal was not or only to a small extent observed in the scans obtained in anaesthetized animals. Such negative BOLD signals located in regions surrounding the activated area are commonly observed in human as well as animal fMRI experiments^26–29^. The exact nature of this effect is still not fully characterized. Contradictory reports are available that designate the negative responses to decreased neural activation on the one hand^27–29^ or to a purely vascular effect caused by active blood redistributions with no neural correlate, on the other hand^26^. The latter is supported by the fact that in normal physiological state, total blood flow in the brain remains constant implying a compensatory redistribution of cerebral blood flow (CBF) when increased flow is needed to a specific activated area (‘blood stealing’)^30^. Smith and colleagues, however, observed that, upon visual stimulation, a negative BOLD response emerges in specific parts of the visual cortex and attributed this effect to attention modulation rather than to a vascular effect. They showed that when focusing on a pattern against a blank background, the pattern attracts the observer’s attention, causing the observer’s attentional resources that were previously spatially diffuse to become focused at one location, resulting in a reduction of attention-related neural activity in other locations within the visual cortex^28^. Based on these findings, negative BOLD signals are most likely caused by a combination of vascular and neuronal effects. Additionally, we must note that negative BOLD signals in different brain regions, species, and stimulation protocols might have different origins. The negative activations we observed in the zebra finch brain in this study are probably not due to attention modulation as they (1) occupy large parts of the brain surrounding, but not including, the auditory forebrain and (2) this pattern did not differ depending on the stimulus to which the animal was exposed (behaviourally relevant song versus synthetic pure tones). In addition, we could co-localize the regions with the most pronounced negative BOLD signal with large blood vessels near the cerebellum, which is a finding in favour of the blood redistribution theory. However, to confirm this, more analyses are necessary such as a complete characterization of the supplying vasculature of the auditory forebrain to ensure that the vascular beds, which co-localize with the regions where negative BOLD signals were detected, are indeed supplying the auditory regions. The observation that anaesthetic agents like medetomidine and isoflurane, which affect the brain’s vasculature resulting in a decreased cerebrovascular reactivity, modulate the negative BOLD signal observed in awake animals, also supports the vascular theory as being the most probable biological mechanism underlying the current observations.

The observation that the negative BOLD was also affected in the Med/Iso group compared to the awake animals, points to a widespread vascular effect induced by this anaesthesia protocol which was comparable to the effect seen for isoflurane alone. In mice, the Med/Iso combination was reported to induce limited vascular effects^15^. Yet, our results do not support this theory in zebra finches. Most probably vascular effects of anaesthesia are strongly dependent on the species so that effects seen in one cannot be directly translated to another model system. Additionally, the dose used in this study might not have been the ideal combination to perfectly counterbalance the vasoconstrictive effect of medetomidine with the vasodilatative effect of the isoflurane. In summary, our results indicate that the Med/Iso combination used in the current study to induce light anaesthesia levels in zebra finches does not ensure stable anaesthesia with limited head motion during scanning and does not limit the vascular effects of the anaesthetics. Yet, we must note that our results do not exclude that alternative combinations (doses) of the anaesthetics can be found that do parallel the advantages of the Med/Iso combination for fMRI experiments as seen in mice.

From previous reports in humans and animals, and observations that anaesthetics depress evoked neural activity and influence cerebrovascular reactivity^31^, one would expect a lower BOLD response amplitude in anaesthetized versus awake conditions. This effect was, however, not observed in the current study. One reason we may not have observed such a lower response is that, at low doses of anaesthesia, the modulation of BOLD response amplitude is small and could thus not be detected due to the limited sensitivity of the Spin Echo (SE) sequence used here^21^. Besides a limited sensitivity of the technique and the use of low doses of anaesthesia, the discrepancy in our results versus previous reports could also be caused by the influence of arterial partial pressure of carbon dioxide and oxygen (pCO_2_ and pO_2_) on the BOLD response in awake versus anaesthetized conditions^32^. In the experimental setup for anaesthetized conditions, inhaled air through the beak mask was supplemented with O_2_ whereas in the awake conditions air in the beak mask was not artificially circulated nor supplemented with O_2_. Instead, the animals inhaled atmospheric air which might have contained a relatively higher concentration of CO_2_ caused by possible limited circulation of fresh air in the confined space of the beak mask. As such, differences in pCO_2_ and pO_2_ in the anaesthesia setup versus the awake setup could have caused a discrepancy between expected and actual detected BOLD response amplitudes in the different conditions. To confirm this, information on the actual pO_2_/pCO_2_ levels during the scans acquired in this study is needed. However, due to practical limitations of the zebra finch scan setup, this information was not collected. Consequently, we recommend to – in addition to monitoring body temperature and breathing rate – include pCO_2_/pO_2_ measurements in future functional imaging experiments and ensure comparable levels within and between groups.

In the last part of this study, we verified whether neural selectivity for natural sounds is influenced by low anaesthesia. There are already numerous reports available on the effects of anaesthesia on auditory processing in songbirds, but the results are less than consistent: some studies have demonstrated an influence of anaesthesia on tuning properties of neurons in primary and secondary auditory regions^9,33^, whereas other studies did not detect any effect of anaesthesia^34–36^. The heterogeneity of these reports is most likely caused by differences in the technique, anaesthesia, and auditory stimuli used as well as the region under study. As different anaesthetics induce their sedative effects in different ways, it is not surprising that their effects on neural tuning vary. Isoflurane, for example, induces a general inhibition by modulating neurotransmitter-gated ion channels, including the γ-aminobutyric acid (GABA) receptor. In contrast, medetomidine acts as an α2-adrenoreceptor agonist which has been shown to increase the release of norepinephrine (NE)^9^. Through NE, medetomidine can have a direct influence on neural selectivity in the auditory forebrain^20,37^. We did not observe a difference in auditory selectivity in isoflurane or Med/Iso anaesthesia versus the awake state. Although, the graphs in Fig. 5 suggest larger differences in BOLD responses to natural versus synthetic pure tones and thus higher sensitivity in the awake state, the general trends were similar for all conditions. In line with previous reports, we found selectivity for complex vocalizations (conspecific song, heterospecific song) versus behaviourally irrelevant synthetic tones both in primary and secondary auditory forebrain regions^38,39^. The data also hinted at a selectivity for conspecific song over heterospecific song in the left auditory forebrain, which is in line with observations made in a previous fMRI study^11^.

In summary, our study is the first to demonstrate the feasibility of fMRI in awake zebra finches. Using an acclimation procedure to accustom the animals to total restraint and to the scanner noise, we were able to scan the birds successfully during wakefulness with minimal head motion resulting in high quality fMRI scans and the detection of reproducible BOLD responses to sound. This finding opens a wide range of new avenues for the investigation of brain function in this animal model. For studies in which the use of anaesthesia is preferred, we showed that, out of the two light anaesthesia protocols that were tested, isoflurane is the most promising for fMRI studies given its high success rate, non-invasive induction and quick recovery. Additionally, we found no effects of light anaesthesia on the selective BOLD responses to natural versus synthetic sounds. Further, our results indicate that the main influence of light anaesthesia on the BOLD response is likely caused by the effect of the anaesthetics on cerebrovascular reactivity, resulting in changes in the extent of positive and negative BOLD responses. In order to validate and characterise this observation more thoroughly, further research on zebra finch brain vasculature and how it is influenced by anaesthesia must be performed.

## Materials and Methods

### Subjects

In this study, we used 28 adult male zebra finches (*Taeniopygia guttata*; >120 days old), which were purchased at a local supplier or bred in the local animal facility. The birds were kept in large indoor aviaries or in group cages (per three; during acclimation) on a 12 hour light/12 hour dark photoperiod and provided food and water *ad libitum*. All experimental procedures were in agreement with the Belgian laws on the protection and welfare of animals and approved by the Committee on Animal Care and Use at the University of Antwerp (license number: 2013–64).

### Acclimation procedure for fMRI in awake conditions

We scanned a subset of the birds in this study in awake conditions (n = 12). To limit head motion during scanning, birds were restrained with a head fix and an adapted body restraining jacket. The jacket covered almost the entire body of the animal leaving their head, neck and tail free. We were able to adjust the size of the jacket with Velcro bands to ensure a tight restraining and as such prevent the birds from opening their wings or moving their body excessively. The head fix consisted of a beak mask and a head fixation tape. The beak mask was similar to the one used for scanning in anaesthetized conditions to ensure comparable head positioning for all scans acquired in this study. In order to reduce stress during scanning in restrained conditions, we gradually acclimated the animals to restraining and scanner noise prior to awake scanning. A schematic overview of the acclimation procedure for awake scanning is shown in Fig. 1. The protocol was based on a protocol that was previously applied to acclimate pigeons to be scanned in awake conditions^16^ and consisted of 3 consecutive phases: (1) Acclimate to body restraining in a dark scanner-like environment; (2) Acclimate to total restraint including head fixation; (3) Acclimate to total restraint and scanner noise. The entire acclimation procedure was performed in the dark in a mock scanner setup located in a laboratory within the imaging centre. We recorded scanner noises of the different sequences included in the planned imaging protocol and subsequently played them back to the birds during acclimation at predefined sound levels through speakers placed at both sides of the mock scanner. The maximal sound level at which the noise was played was equal to the real sound level measured in the middle of the scanner bore (e.g. for the fMRI sequence, the real sound level of the scanner noise was around 68 dB). After 25 consecutive days of acclimation, the birds were ready to be scanned in restrained conditions for up to two hours. We did not allow any gap days during the entire acclimation period and or between the end of the acclimation period and the day of scanning. In case scanning could not be performed the day immediately following the end of the acclimation period, the acclimation period was extended to avoid any gap days. After each acclimation or awake scanning session, we rewarded the birds with an apple treat. We must however note that not all birds payed attention to this treat.

### Anaesthesia and animal monitoring

Besides scans in awake conditions, we also acquired fMRI scans under Med/Iso and isoflurane anaesthesia. All birds included in the awake experiment were also scanned under isoflurane anaesthesia (n = 12). Additionally, we scanned a separate subgroup of birds (n = 12) both under isoflurane and under Med/Iso anaesthesia. The order of the different sessions per individual bird was randomized over birds with an interval of at least one week between the individual sessions. Four additional birds could only be scanned under one anaesthesia condition (one bird isoflurane and three birds Med/Iso). The total group size per condition was thus 12 birds for the awake condition, 15 birds for the Med/Iso anaesthetized condition and 25 birds for the isoflurane anaesthetized condition (of which 12 were the same birds as in the awake group and another 12 the same as in the Med/Iso group).

Similar to the fMRI study by Boumans^9^, we administered medetomidine anaesthesia in the pectoral muscle. The birds initially received a bolus of 0.15 mg/kg medetomidine with 5 mg/kg ketamine. Medetomidine anaesthesia was combined with administration of 2.5% Isoflurane (IsoFlo®, Abbott, Illinois, USA) relative to the carrier gas (mixture of oxygen and nitrogen at flow rates of 100 and 200 cm³/min, respectively) through the beak mask fixed to the scanner bed. Within approximately 15 minutes after this induction stage, we gradually decreased the concentration of isoflurane to 0.4% which was maintained throughout the rest of the experiment. Ten minutes after bolus injection, we initiated a continuous infusion of medetomidine at a rate of 0.3 mg/kg/h through a catheter in the pectoral muscle. For the scans under only isoflurane anaesthesia, induction was performed at 2.5% isoflurane. Subsequently, the concentration was gradually decreased within approximately 15 minutes after induction to a final concentration of 1.2% (relative to the oxygen/nitrogen mixture used as carrier gas) maintained for the rest of the experiment. Throughout the entire imaging protocol, the physiological condition of the animals was continuously monitored by means of a small pneumatic sensor for the breathing rate and a temperature-sensitive cloacal probe for the body temperature (MR-compatible Small Animal Monitoring and Gating system, SA Instruments, Inc.). The latter was connected to a tightly controlled warm air feedback system to maintain the birds’ body temperature within narrow physiological ranges (40.0 ± 0.1) °C.

### Auditory stimuli

During fMRI, we exposed the birds to three types of auditory stimuli: (i) PT, (ii) unfamiliar CON, and (iii) unfamiliar HET. CON and HET stimuli were reused from a previous fMRI study^11^. Briefly, CON stimuli consisted of several individual songs recorded from a single adult male bird interleaved with silence periods of 0.5 seconds. In total, six different CON stimuli each consisting of songs from a different male were used and randomly assigned to the different fMRI sessions acquired in this study. HET stimuli corresponded to songs of two European starlings (*Sturnus vulgaris*) and one canary (*Serinus canaria*). PT stimuli consisted of pure tones at different frequencies between 1 and 7 kHz with a duration of 0.7 seconds interleaved with silence periods of 0.1 to 0.2 seconds. The total length of each stimulus was 16 s. We applied an equalizer function to the stimuli before presenting them in the scanner to compensate for the artificial frequency dependent increase of sound intensity by the scanning setup (see^22^ for details on this procedure).

### MR Imaging

#### Auditory fMRI

We performed functional imaging on a horizontal 7 Tesla MR system (Pharmascan 70/16 US, Bruker Biospin, Germany) following established protocols^22^. Briefly, fMRI scans consisted of a time series of 298 T_2_-weighted rapid acquisition relaxation-enhanced (RARE) volumes (echo time/ repetition time: 60/2000 ms) and each volume was composed of 15 sagittal slices covering the whole brain (slice thickness = 0.75 mm, interslice gap = 0.05 mm) with an in-plane resolution of (0.25 × 0.25) mm² (acquisition matrix: 64 × 32, image matrix 64 × 64). The acquisition time per volume was 8 s. Besides the functional scans, we acquired a high-resolution anatomical 3D RARE image (resolution = (0.07 × 0.07 × 0.07) mm³) for each bird in the same orientation as the fMRI scans to facilitate subsequent spatial normalization of images.

All scans in both awake and anaesthetized conditions were performed in the dark. During the awake scans, we did not notice any obvious decreases in breathing rate which could indicate that the birds were falling asleep during scanning. Additionally, artefacts at the level of the eyes caused by eye blinking were often observed in the fMRI time series which also indicates that the birds were really awake during scanning. In anaesthetized conditions, the functional scans were not acquired earlier than 30 minutes after induction of the anaesthesia. Auditory stimulation was played back at a mean intensity (in terms of root mean square) of 70 dB through dynamic loudspeakers (Visation, Germany; magnets removed), placed at each side of the bird’s head. The different stimulus types were presented randomly to the birds in an ON/OFF blocked design, alternating 16 seconds of stimulation (ON period) and 16 seconds of rest (OFF period). A session consisted of 72 ON blocks (24 per stimulus type) and 72 OFF blocks. During each block, we acquired two fMRI volumes resulting in 48 images per stimulus type and per subject.

#### Angiography

Four birds underwent an extra imaging session to obtain a high-resolution angiogram of the entire brain. We acquired the angiograms on a 9.4 T small animal MR system (Bruker Biospin, Germany) equipped with a receive-only cryo-probe designed for a mouse head (Bruker Biospin, Germany), using a fast low angle shot (FLASH) sequence (imaging parameters: echo time/repetition time: 3.4/17 ms; matrix (320 × 320); in plane resolution (0.0625 × 0.0625) mm²; number of slices: 80; slice package extent: 12 mm; averages: 3; slice package orientation: horizontal). Immediately after the angiogram, we acquired an anatomical T2-weighted RARE scan with the same geometry as the FLASH to enable spatial registration of the angiogram to a population-based template. During this imaging session, isoflurane (induction 2.5%, maintenance 1.5%) was used to anaesthetize the zebra finches.

### Data processing and analysis

We performed both data pre-processing and statistical voxel-based analyses using the Statistical Parametric Mapping toolbox (SPM12, Wellcome Trust Centre for Neuroimaging, London, UK; http://www.fil.ion.ucl.ac.uk/spm). The statistical analyses of the ROI-based data was performed in JMP® (Version 13, SAS Institute Inc., Cary, NC, 1989–2007). The data processing procedures were similar to those previously described^22^. In short, the functional data were realigned and subsequently co-registered to their corresponding 3D RARE image. A population based template was generated from the high quality anatomical 3D RARE images acquired in this study (ANTs (http://stnava.github.io/ANTs/)). Spatial normalization of all scans to this template enabled between-subject comparisons^40^. Finally, to achieve in-plane smoothing, we applied a Gaussian kernel of 0.5 mm full width at half maximum (FWHM).

We applied a high pass filter on the functional data (352-seconds cut-off period) to remove low frequency drifts in the BOLD signal. For the first level analysis, we subsequently modelled for each subject the BOLD responses as a box-car function convolved with a canonical hemodynamic response function within the framework of the general linear model (GLM) to analyse brain activation differences related to playback of the different stimuli. We included the six estimated movement parameters derived from the realignment corrections as regressors in the model to account for the residual effects of head motion. After the estimation of the GLM parameters (β), we calculated different *t*-contrast images (containing weighted parameter estimates) for different comparisons including all stimuli vs rest, CON vs rest, HET vs rest and PT vs rest.

### ROI based analysis

From the single subject contrast maps for all stimuli vs rest, we first extracted the size, peak *T*, and mean *T* from the activated cluster (all stimuli > rest) within the auditory forebrain encompassing the regions Field L, NCM and CMM (threshold *p*_uncorrected_ < 0.01) and assessed differences in these parameters between the conditions. For this, we used a linear mixed model with condition as independent variable and bird ID as random variable. Tukey’s HSD (honest significant difference) test was applied for all post hoc tests with α < 0.05 unless otherwise noted.

### Voxel-based group analysis

A two-way mixed-effect ANOVA was used to test for the interaction between condition and stimulus as well as for the main effect of stimulus. For this, we constructed a 3 × 3 factorial design including condition (awake, isoflurane, Med/Iso) as a between-subject factor and stimulus (PT vs rest, CON vs rest, HET vs rest) as a within-subject factor. We applied a threshold of *p*_uncorrected_ < 0.05 for visualization of the results and only clusters of at least 53 voxels were considered significant (Monte Carlo simulation: α < 0.05^17^; performed using AlphaSim (AFNI)^41^). In addition, we only included clusters which co-localized with the auditory forebrain in our analysis.

To explore the main effect of condition, a separate whole brain one-way Anova analysis was applied to compare BOLD responses to sound (all sounds > rest) between the different conditions. We applied a threshold of *p*_uncorrected_ < 0.001 for visualization of the results only clusters with at least 12 voxels were considered significant (Monte Carlo simulation: α < 0.05).

For all post-hoc *t*-tests within the main effects or the interaction, a threshold of *p* < 0.05 was applied with FWE correction to account for errors induced by multiple comparisons. Reflecting the voxel basis of the analysis, results of these tests are reported by the highest voxel *T* value within each cluster (*T*_max_) and the associated voxel *p* value.

### Analysis of head motion

The six estimated head motion parameters from the realignment step in the pre-processing of the fMRI time series, were used to estimate head motion. From these parameters, we extracted for each animal, in each condition, the maximal head translation and rotation. Additionally, the six motion parameters were condensed to a single vector representing the displacement following the rigid transformation for a voxel at location ((x, y, z) = (1, 1, 1) mm) to obtain a measure of total head motion comparable between animals. This parameter was subsequently used to calculate the CV of the total head motion over time. For statistical analysis of the differences in head motion we used a mixed-effects model with condition as independent variable and bird ID as random variable. Tukey’s HSD test was applied for all post hoc tests with α < 0.05 unless otherwise noted.

### Analysis of breathing rate

For the animals scanned awake and under isoflurane anaesthetized condition, an overall range for the breathing rate (minimum–maximum values) over the entire fMRI scan was registered. For the remaining subjects scanned under isoflurane and Med/Iso anaesthesia, we registered the breathing rate at fixed times during the fMRI acquisition (every 3 minutes approximately), which allowed for a more accurate measure of average breathing rate and of the variation in breathing rate over time. We used mixed-effects models with condition as independent variable and bird ID as random variable to test for differences in average breathing rate (awake vs isoflurane vs Med/Iso) and of the CV (repeated measures isoflurane vs Med/Iso). Tukey’s HSD test was applied for all post hoc tests with α < 0.05 unless otherwise noted. All data are presented as mean ± SD unless otherwise noted.

### Data availability

The datasets generated and analysed within the current study are available from the corresponding author upon request.

## Electronic supplementary material

Supplementary Information
