# Supplementary material for: Auditory evoked BOLD responses in awake compared to lightly anaesthetized zebra finches
Source: Sci Rep. 2017 Oct 19;7:13563. doi: 10.1038/s41598-017-13014-x (PMC5648849; doi:10.1038/s41598-017-13014-x)
Supplement: Supplementary file 1 — Supplementary Information [file 41598_2017_13014_MOESM1_ESM.pdf]

AUDITORY EVOKED BOLD RESPONSES IN AWAKE COMPARED TO LIGHTLY ANAESTHETISED ZEBRA FINCHES

Van Ruijssevelt L.[1], Hamaide J.[1], van Gurp M.T.[1], Verhoye M.[1], and van der Linden A.[1]

[1] Bio-Imaging lab, Department of Biomedical Sciences, University of Antwerp, 2610 Antwerpen, Belgium

Supplementary information

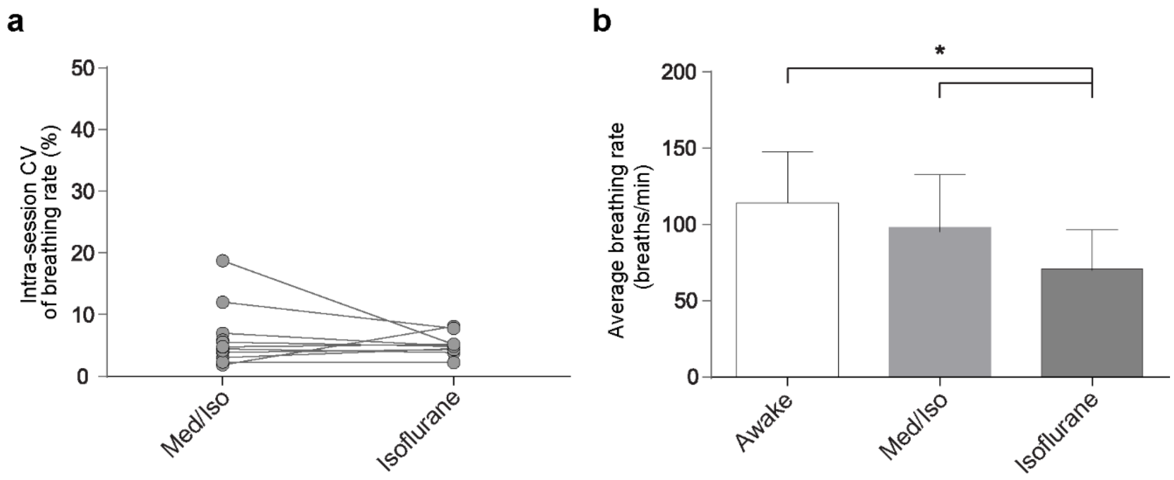

Supplementary figure S1. Effect of light anaesthesia on breathing rate. (a) intra-session coëfficient of variation (CV) of the breathing rate for animals measured under Med/Iso and isoflurane anaesthesia (n=10); (b) Average breathing rate (± SEM) for subjects scanned in awake (n=12), Med/Iso anaesthetized (n=14), and isoflurane anaesthetized conditions (n=25). (*$p<0.05$)

Supplementary table S2. Overview of the group sizes and of the success rate of the functional scans per group.

	Awake	Isoflurane	Med/Iso
**Subj1**	successful	successful	
**Subj2**	successful	successful	
**Subj3**	successful	successful	
**Subj4**	successful	successful	
**Subj5**	successful	successful	
**Subj6**	successful	successful	
**Subj7**	successful	successful	
**Subj8**	successful	successful	
**Subj9**	successful	successful	
**Subj10**	successful	successful	
**Subj11**	training unsuccessful	successful	
**Subj12**	training unsuccessful	successful	
**Subj13**		no clear BOLD response in Field L	
**Subj14**		successful	excessive head motion
**Subj15**		successful	excessive head motion
**Subj16**		successful	excessive head motion
**Subj17**		successful	persistently woke up
**Subj18**		successful	no clear BOLD response in Field L
**Subj19**		successful	no clear BOLD response in Field L
**Subj20**		successful	successful
**Subj21**		successful	successful
**Subj22**		successful	successful
**Subj23**		successful	successful
**Subj24**		no clear BOLD response in Field L	successful
**Subj25**		no clear BOLD response in Field L	successful
**Subj26**			successful
**Subj27**			successful
**Subj28**			successful
